# Lifetime prevalence of suicidal ideation among men who have sex with men: a meta-analysis

**DOI:** 10.1186/s12888-017-1575-9

**Published:** 2017-12-21

**Authors:** Zhenzhou Luo, Tiejian Feng, Hanlin Fu, Tubao Yang

**Affiliations:** 1 Department of Epidemiology and Health Statistics, Xiangya School of Public Health, Central South University, Hunan, China; 2 Shenzhen Nanshan Center for Chronic Disease Control, Guangdong, China; 3 Shenzhen Center for Chronic Disease Control, Guangdong, China

**Keywords:** Suicidal ideation, Men who have sex with men, Prevalence, Meta-analysis

## Abstract

**Background:**

Suicide is a leading cause of death among men who have sex with men (MSM) and suicidal ideation may put individuals at higher risk of suicide. A great disparity of lifetime prevalence of suicidal ideation among MSM was observed across studies, indicating the importance of a reliable estimation of the pooled lifetime prevalence. However, the only one published meta-analysis estimating the pooled lifetime prevalence of suicidal ideation among MSM was conducted in 2008 with only 2 eligible studies. Subsequently, there was a rapid increase of publications about lifetime suicidal ideation among MSM, suggesting that an update on the pooled lifetime prevalence of suicidal ideation among MSM was necessary. Therefore, this study aimed to update the estimation of the pooled lifetime prevalence of suicidal ideation among MSM.

**Methods:**

Electronic databases of PubMed, CINAHL, Scopus (social science), Embase and PsycInfo were searched until September 2017 to identify relevant studies. Cross-sectional studies exploring the lifetime prevalence of suicidal ideation among MSM were enrolled. Heterogeneity was evaluated using the Cochran *Q* test and quantified using the *I* ^2^ statistic. The possibility of publication bias was assessed using both Begg’s rank test and Egger’s linear test, and an Egger’s funnel plot for asymmetry was presented. Subgroup analyses were performed according to the geographic area, sample source and HIV status.

**Results:**

Nineteen studies with a total of 26,667 MSM were included, of which 9374 were identified with suicidal ideation. A high degree of heterogeneity (*P* ≤ 0.001, *I* ^2^ =99.2%) was observed among the eligible studies, with the reported prevalence ranging from 13.18 to 55.80%. The pooled lifetime prevalence of suicidal ideation among MSM by a random effects model was 34.97% (95% confidence interval: 28.35%–41.90%). Both the Begg’s rank test and Egger’s linear test indicated low possibility of publication bias. Subgroup analyses showed that the lifetime prevalence of suicidal ideation among MSM differed significantly by geographic area, sample source and HIV status (*P* < 0.05).

**Conclusions:**

The high pooled lifetime prevalence of suicidal ideation among MSM found in this meta-analysis significantly underscores the importance of early assessment of suicidal ideation among MSM, as well as the need for strengthening the psychological interventions.

**Electronic supplementary material:**

The online version of this article (10.1186/s12888-017-1575-9) contains supplementary material, which is available to authorized users.

## Background

Suicide, a leading cause of death among men who have sex with men (MSM), has become a public health issue [1]. In Canada, more MSM are estimated to have died from suicide than from HIV-related illness in 2011 [1], and in China, a rapid increase of suicide-related behaviors among MSM has been frequently reported [2–4]. Suicidal ideation, defined as thinking about, considering or planning for suicide [5], may lead to higher risk of suicide [6, 7]. In a review by David Klonsky et al., it has been suggested that an important reason for the limited achievement in reducing suicide is inadequate knowledge, especially about why and when suicidal ideation progresses to potentially suicidal attempts [6]. Additionally, the risk for suicide is significantly higher in those with suicidal ideation than those without suicidal ideation [7]. Furthermore, compared with heterosexual males, MSM are at higher risk for suicidal ideation [8, 9], which may be associated with the discrimination and stigma brought by their sexual orientation [8, 10, 11]. For example, in a worldwide population-based study, Mathy found that MSM in South America, North America and Asia were at 7.5, 2.1 and 2.9 times higher risk for suicidal ideation than heterosexual males, respectively [12]. Based on these findings, the assessment of suicidal ideation is imperative among MSM.

Numerous studies have estimated the lifetime prevalence of suicidal ideation among MSM. However, the reported prevalence varied greatly across studies, ranging from 10.6 to 55.3% [13, 14], indicating the importance of a reliable estimation of the pooled lifetime prevalence of suicidal ideation among MSM, which could help the service providers to identify the accurate amount of those with suicidal ideation and hence implement appropriate interventions to reduce suicide. Literature search showed that there was one published meta-analysis which estimated the pooled lifetime prevalence of suicidal ideation among MSM [15]. However, the meta-analysis included exclusively studies with a concurrent heterosexual comparison group, and only 2 studies were included when estimating the pooled lifetime prevalence, which significantly lowered the generalizability of their findings. Furthermore, the meta-analysis was conducted in 2008 and, subsequently, there was a rapid increase of publications about lifetime suicidal ideation among MSM, suggesting that an update on the pooled lifetime prevalence of suicidal ideation among MSM was necessary. Therefore, this study aimed to update the estimation of the pooled lifetime prevalence of suicidal ideation among MSM.

In addition, previous studies have indicated that suicide-related behaviors among sexual minorities differed with different sample sources and HIV statuses [11, 16]. For example, in a study by Hottes et al., it was found that the pooled lifetime prevalence of suicidal attempt in lesbian, gay and bisexual (LGB) individuals for population-based and community-based surveys was 11 and 20%, respectively [16], and HIV-positive MSM were at higher risk of suicidal ideation than HIV-negative MSM [11]. Also, the prevalence of suicidal ideation among MSM may differ by geographic area, which could be explained by the differences in socio-demographic factors and economical levels, as well as the cross-cultural differences in the attitude towards sexual minorities [12]. For example, it has been estimated that the lifetime prevalence of suicidal ideation among MSM in North America and South America was 22.6 and 34.6%, respectively [12]. Therefore, the pooled lifetime prevalence stratified by geographic area, sample source and HIV status was also explored in this study.

## Methods

### Search strategy

This meta-analysis was based on the Preferred Reporting Items for Systematic Reviews and Meta-Analyses (PRISMA) guidelines (the Additional file 1). Electronic databases of PubMed, CINAHL, Scopus (social science), Embase and PsycInfo were searched until September 2017. Subject headings were used where possible; otherwise text words were used. Consistent with previous meta-analyses on the MSM population [17–19], MSM were identified by using a broad set of text words in this meta-analysis, including MSM, men who have sex with men, homosexual, homosexuality, bisexual, bisexuality and gay. Specifically, for the databases of Embase and PsycInfo, a combination of the subject headings “suicidal ideation” and “male homosexuality” was used, while for the databases of PubMed, CINAHL and Scopus, the search terms were “((lifetime[Text Word]) AND suicid*[Text Word]) AND (((((((homosexuality[Text Word]) OR bisexuality[Text Word]) OR gay[Text Word]) OR bisexual[Text Word]) OR MSM[Text Word]) OR men who have sex with men[Text Word]) OR homosexual[Text Word])”. The reference lists of four reviews about suicide-related behaviors in sexual minorities [15, 16, 20, 21] and all included studies were hand searched for further relevant articles.

### Eligibility criteria

Articles were included in this meta-analysis if they fulfilled the following criteria: (1) the study design of the article was cross-sectional; (2) the target population of the article focused on or included men who have had sexual intercourse with men, and/or men who have been sexually attracted to men [22, 23]; (3) the article employed a clear definition of suicidal ideation with a binary response of “yes” or “no”; (4) the article provided information on the sample size and lifetime prevalence of suicidal ideation; (5) the article was published in a peer-reviewed journal in English language. Articles were excluded if: (1) they specifically targeted only participants who sought mental health services; (2) they specifically targeted only youths or adolescents, though age was not a restriction in this meta-analysis; (3) they were review, book chapter, case-report or comment. Additionally, if repeated data were observed between different articles, only the earlier publication was included.

### Data extraction

Two investigators extracted relevant data independently and carefully, and any inconsistencies between them were further discussed and resolved by consensus. The outcome of this meta-analysis was the lifetime prevalence of suicidal ideation. Though suicidal attempt is considered better proxies for suicide death than suicidal ideation [16], knowledge about why and when suicidal ideation progresses to suicidal attempts remains inadequate, indicating the importance of the estimation of lifetime prevalence of suicidal ideation.

For all included studies, the following data were extracted: first author, year of publication, location of study, sample source, sampling method, number of participants with suicidal ideation, sample size, and quality of study. Additionally, if available, data on geographic area and HIV status were also extracted. Consistent with previous meta-analyses, the geographic area was categorized into high-income countries, and low- and middle- income countries, and the sample source was categorized into population-based and community-based [16, 24]. Specifically, population-based surveys involved predominantly heterosexual individuals and community-based surveys involved exclusively MSM from the venues that MSM visits (e.g., gay bars, clubs, and MSM Web sites).

### Quality assessment

Consistent with previous meta-analyses on cross-sectional studies, the Agency for Healthcare Research and Quality (AHRQ) was used to assess the quality of eligible articles [25, 26]. It is an 11-item scale with Yes/No/Unclear response options. This scale focuses on assessing the quality of reporting, with most of the items assessing whether or not relevant information was reported. The response of “Yes” for each item was scored “1”, and the response of “No” or “Unclear” for each item was scored “0”. A total score of 0 to 3, 4 to 7, and 8 to 11 indicates low, moderate, and high quality, respectively.

### Statistical analysis

All statistical analyses for this study were performed using the statistical software R version 3.4.1. Heterogeneity was assessed using the Cochran  *Q* test and quantified using the *I* ^2^ statistic. The pooled lifetime prevalence of suicidal ideation among MSM was estimated using Freeman-Tukey transformation of inverse hyperbolic sine function method by a random effects model when significant heterogeneity (*P* ≤ 0.10, *I* ^2^ >50%) was observed. Otherwise, a fixed effects model was used [27]. Sensitivity was assessed by the effect of excluding articles with low quality on the stability of the pooled prevalence [28, 29]. Publication bias was evaluated using both Begg’s rank test and Egger’s linear test, and an Egger’s funnel plot for asymmetry was presented [30, 31]. Subgroup analyses were performed according to the geographic area, sample source and HIV status. The *χ* ^2^ test was used to assess the difference across subgroups [28, 32, 33] and the significant level was set at *P* < 0.05.

## Results

### Search results

**Fig. 1:**
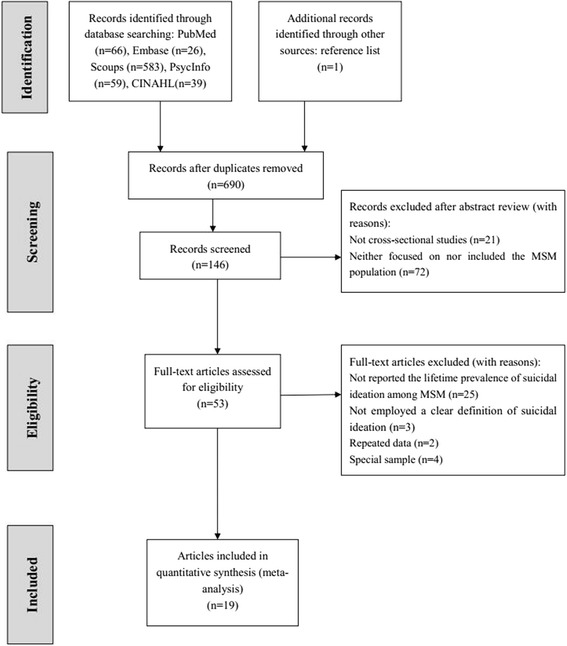
Flow chart of study identification and selection

A total of 774 articles were initially yielded for this meta-analysis. Of these, 53 full articles were shortlisted for the eligibility assessment. Among the 53 articles, 25 were excluded for not reporting the lifetime prevalence of suicidal ideation among MSM, 3 were excluded for not employing a clear definition of suicidal ideation, 2 were excluded for repeated data, and 4 were excluded for targeting only youths or adolescents or only participants who sought mental health services. Finally, 19 articles were included in this meta-analysis (Fig. 1).

### Study characteristics

The characteristics of eligible studies are presented in Table 1. A total of 26,667 participants were involved, of which 9374 were identified with lifetime suicidal ideation. Among the 19 eligible studies, 11 were conducted in high-income countries (including USA, Canada, United Kingdom, Netherlands, Switzerland, and Estonia) and 7 were conducted in low- and middle- income countries (including Lao People’s Democratic Republic, Nepal, China, Uganda, Gambia, Togo, and Burkina Faso); 6 were population-based surveys and 13 were community-based surveys; 7 used random sampling method and 7 used snowball sampling method and/or respondent-driving sampling method; furthermore, according to the AHRQ scale, 17 were identified with moderate quality, and 2 were identified with low quality (the Additional file 2).

**Table 1:** Characteristics of the studies included in this meta-analysis

First author	Year of publication	Location of study	Sample source	Sampling method	Number of MSM with suicidal ideation	Sample size	Quality of study
Cochran [49]	2000	USA	Population-based	Random sampling	32	78	Moderate
Mathy [12]	2002	Worldwide	Population-based	Internet	878	3887	Moderate
Botnick [50]	2002	Canada	Community-based	Not reported	150	345	Moderate
Warner [51]	2004	United Kingdom	Community-based	Snowball sampling	357	741	Moderate
de Graaf [8]	2006	Netherlands	Population-based	Random sampling	33	82	Moderate
Sheridan [52]	2009	Lao People’s Democratic Republic	Community-based	Venue-day-time sampling	90	540	Moderate
Brennan [53]	2010	Canada	Population-based	Random sampling	268	948	Moderate
Wang [14]	2012	Switzerland	Community-based	Random sampling	316	571	Moderate
Deuba [54]	2013	Nepal	Community-based	Snowball sampling	159	339	Moderate
Wang [34]	2013	Switzerland	Community-based	Random sampling	154	276	Moderate
Chen [55]	2015	China	Community-based	Snowball sampling	398	1530	Low
Ferlatte [56]	2015	Canada	Community-based	Internet	4180	8382	Low
Parker [57]	2015	Estonia	Community-based	Internet	118	265	Moderate
Blosnich [58]	2016	USA	Population-based	Random sampling	269	756	Moderate
Hottes [59]	2016	Canada	Population-based	Random sampling	1289	4675	Moderate
Mu [47]	2016	China	Community-based	Respondent-driven sampling	148	807	Moderate
Hladik [60]	2016	Uganda	Community-based	Respondent-driven sampling	270	608	Moderate
Stahlman [10]	2016	Gambia, Togo, Burkina Faso	Community-based	Respondent-driven sampling and snowball sampling	205	1555	Moderate
Kohlbrenner [61]	2016	Nepal	Community-based	Respondent-driven sampling	60	282	Moderate

### Pooled lifetime prevalence of suicidal ideation among MSM

**Fig. 2:**
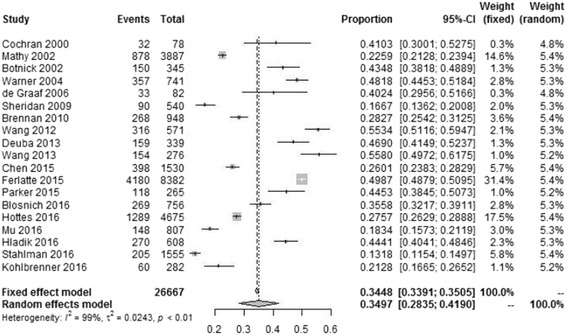
Forest plot presenting the lifetime prevalence of suicidal ideation among MSM

The lifetime prevalence of suicidal ideation reported in eligible studies ranged from 13.18% [10] to 55.80% [34], with the lowest reported in three West African countries (including Gambia, Togo and Burkina Faso) and the highest in Switzerland. Significant heterogeneity (*P* ≤ 0.001, *I* ^2^ =99.2%) was observed among the eligible studies and a random effects model was used to estimate the pooled lifetime prevalence. The pooled lifetime prevalence of suicidal ideation among MSM was 34.97% (95% confidence interval (CI): 28.35%–41.90%). Figure 2 shows the details.

### Sensitivity analysis and publication bias

After excluding 2 articles with low quality, the pooled lifetime prevalence of suicidal ideation decreased slightly from 34.97% (95% CI: 28.53%–41.90%) to 34.61% (95% CI: 28.67%–40.79%), suggesting low sensitivity of this meta-analysis.

**Fig. 3:**
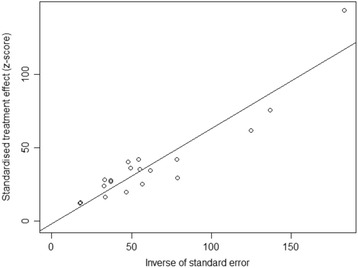
Egger’s funnel plot of the 19 included studies in this meta-analysis

In addition, both the results of Begg’s rank test (z = 1.434, *P* = 0.152) and Egger’s linear test (*t* = −0.392, *P* = 0.700) indicated low possibility of publication bias. Consistent with Egger’s linear test, the Egger’s funnel plot was symmetrical (Fig. 3).

### Subgroup analyses

The results of subgroup analyses are shown in Table 2. The pooled lifetime prevalence of suicidal ideation in high-income countries, and low- and middle- income countries was 42.55% (95% CI: 34.78%–50.50%) and 25.78% (95% CI: 17.33%–35.26%), respectively. The pooled lifetime prevalence in population-based and community-based surveys was 30.58% (95% CI: 26.25%–35.09%) and 36.34% (95% CI: 26.93%–46.32%), respectively. The pooled lifetime prevalence among HIV-positive and HIV-negative MSM was 25.75% (95% CI: 17.64%–34.76%) and 16.99% (95% CI: 3.17%–38.52%), respectively. Besides, the heterogeneity was high in most subgroups. However, the heterogeneity was quite low when estimating the pooled lifetime prevalence of suicidal ideation in HIV-positive MSM (*P* = 0.269, *I* ^2^ =18.0).

**Table 2:** Subgroup analyses of the lifetime prevalence of suicidal ideation among MSM

Subgroup		Number. of studies	MSM with suicidal ideation	Total sample	Pooled prevalence of suicidal ideation (95% CI) (%)	Heterogeneity		Between group difference	
						*I* ^2^ (%)	*P* value	*χ* ^2^	*P* value
Geographic area								613.596	<0.001
	High-income countries	11	7166	17,119	42.55 (34.78–50.50)	98.8	<0.001		
	Low- and middle- income countries	7	1330	5661	25.78 (17.33–35.26)	98.3	<0.001		
Sample source								554.585	<0.001
	Population-based	6	2769	10,426	30.58 (26.25–35.09)	93.8	<0.001		
	Community-based	13	6605	16,241	36.34 (26.93–46.32)	99.2	<0.001		
HIV status								11.504	0.001
	Positive	2	38	149	25.75 (17.64–34.76)	18.0	0.269		
	Negative	3	344	2283	16.99 (3.17–38.52)	99.1	<0.001		

Subgroup analyses also indicated that the lifetime prevalence of suicidal ideation among MSM differed significantly by geographic area, sample source and HIV status (*P* < 0.05). Specifically, the lifetime prevalence of suicidal ideation was significantly higher in high-income countries (vs. low- and middle- income countries), in community-based surveys (vs. population-based surveys) and in HIV-positive MSM (vs. HIV-negative MSM).

## Discussion

This meta-analysis provided an update on the pooled lifetime prevalence of suicidal ideation among MSM. Nineteen eligible studies with a total of 26,667 participants were involved, of which 9374 were identified with suicidal ideation. The reported lifetime prevalence of suicidal ideation ranged from 13.18% [10] to 55.80% [34] among the eligible studies, and the pooled lifetime prevalence of suicidal ideation among MSM by a random effects model was 34.97% (95% CI: 28.35%–41.90%).

The pooled lifetime prevalence of suicidal ideation among MSM found in this study (34.97%) was not only much higher than that among general population found in previous meta-analyses (3.9–11.5%) [35–38], but also higher than the lifetime prevalence of suicidal ideation among lesbians found in some previous studies. For example, in a general population-based sample from the Netherlands, de Graaf et al. found that the lifetime prevalence of suicidal ideation among lesbians was 23.3% [8], and in a Canadian population-based study by Steele et al., this rate was 29.5% [39]. The high pooled lifetime prevalence of suicidal ideation among MSM found in this study, which may be associated with the discrimination, stigma, prejudice and isolation brought by their sexual orientation [8, 10, 11], significantly underscores the importance of early assessment of suicidal ideation among MSM, as well as the need for strengthening the psychological interventions.

Subgroup analysis found that the lifetime prevalence of suicidal ideation in high-income countries was higher than that found in low- and middle- income countries. This tendency was contradictory to a study by Mathy, which showed that the lifetime prevalence of suicidal ideation among MSM was 22.6% in North America and 34.6% in South America [12]. The high heterogeneity observed when estimating the pooled lifetime prevalence stratified by geographic area (*I* ^2^ =98.8% for studies involved in high-income countries and *I* ^2^ =98.3% for studies involved in low- and middle- income countries) may lead to the contradictory results. More cross-cultural studies are warranted to better clarify the difference in the lifetime prevalence of suicidal ideation observed in different geographic areas.

Subgroup analyses also indicated that the lifetime prevalence of suicidal ideation differed significantly between different sample sources. Specifically, MSM from community-based surveys exhibited higher lifetime prevalence of suicidal ideation, in comparison with those from population-based surveys. This finding is consistent with a previous meta-analysis [16]. By pooling 30 cross-sectional studies conducted in North America and Western Europe, Hottes et al. found that the pooled lifetime prevalence of suicidal attempt was 11 and 20% among LGB individuals from population-based and community-based surveys, respectively [16]. Venue-based LGB community surveys may induce selection bias and tended to overrepresent gay or lesbian-identified, urban, and high-income sexual minorities [40], thus contributing to a higher lifetime prevalence of suicide-related behaviors.

Additionally, this meta-analysis found that the lifetime prevalence of suicidal ideation among HIV-positive MSM was much higher than that among HIV-negative MSM, indicating more attention should be paid to HIV-positive MSM. Suicidal ideation may arise as the infected MSM learned of their HIV status [41]. This was mainly due to the fact that compared with HIV-negative MSM, HIV-positive MSM may experience higher levels of stigma associated with increased risk for suicidal ideation [11].

It is noteworthy that none of the eligible studies were identified with high quality according to the AHRQ scale. This was specifically because most eligible studies did not indicate how confounding was assessed and did not summarize the completeness of data collection. Furthermore, none of the eligible studies indicated if evaluators of subjective components of study were masked to other aspects of the status of the participants, and explained how missing data were handled in the analysis. A low level of quality of reporting did not necessarily imply a low level of quality of methodology [42]. Additionally, levels of quality of observational studies may differ by different types of assessment scales [43]. Quality is an amorphous concept and it is important to distinguish between the quality of reporting and the quality of methodology, which reflects the susceptibility to bias and assesses the validity [43]. Nonetheless, reporting detailed information on the methods may help identify the appropriateness of methodology. Thus, it is imperative for the researchers to report their findings of observational studies in accordance with the appropriate guidelines, such as the STROBE checklist.

Certain limitations should be acknowledged in this meta-analysis. First, recall bias might exist when collecting information on the lifetime prevalence of suicidal ideation, as a consequence of which, the misclassification of suicidal ideation among the included studies remains a possibility. Second, the heterogeneity in the whole sample and most subgroups was high. The eligible studies included in this meta-analysis came from a varied range of geo-political background with diverse social and environmental factors, which may lead to the high heterogeneity. Additionally, though numerous studies have indicated that psychological stress (i.e. discrimination, stigma and stressful life events) and mental health problems (i.e. depression and anxiety) may affect the lifetime prevalence of suicidal ideation among sexual minorities [44–46], subgroup analyses stratified by these factors were unable to conduct since quite few eligible studies have reported related information among MSM. Future research should, therefore, explore more factors associated with the lifetime prevalence of suicidal ideation among MSM, especially factors related to psychological stress and adverse mental health outcomes. Besides, though some studies have indicated that age may be associated with suicide-related behaviors [47, 48], subgroup analysis stratified by age was unable to perform due to the inconsistent cutoff points.

Despite the preceding limitations, this study has quite a few strengths. First, this study provided an update on the pooled lifetime prevalence of suicidal ideation among MSM. Nineteen eligible studies conducted globally were involved, which significantly increased the generalizability of our findings. Second, this meta-analysis provided the first quantitatively pooled lifetime prevalence of suicidal ideation among MSM stratified by the subgroups: geographic area, sample source and HIV status. The different prevalence found within each subgroup would be helpful in identifying the factors associated with suicidal ideation. Finally, low sensitivity and low possibility of publication bias significantly increased the reliability of the findings of this study.

## Conclusions

The pooled lifetime prevalence of suicidal ideation among MSM was 34.97% (95% CI: 28.35%–41.90%). For population-based and community-based surveys, the pooled lifetime prevalence was 30.58% (95% CI: 26.25%–35.09%) and 36.34% (95% CI: 26.93%–46.32%), respectively. The lifetime prevalence of suicidal ideation among MSM differed significantly by geographic area, sample source and HIV status. The findings of this meta-analysis significantly underscore the importance of early assessment of suicidal ideation among MSM, as well as the need for strengthening the psychological interventions.

## Additional files

Additional file 1: PRISMA Checklist. Description of data: details of PRISMA Checklist. (DOC 64 kb)

Additional file 2: Quality of the eligible studies. Description of data: details about the quality of the eligible studies. (DOCX 17 kb)

## Abbreviations

AHRQ: Agency for Healthcare Research and Quality
CI: Confidence interval
LGB: Lesbian, gay and bisexual
MSM: Men who have sex with men
PRISMA: Preferred Reporting Items for Systematic Reviews and Meta-Analyses
