# Supplementary material for: Lifetime prevalence of suicidal ideation among men who have sex with men: a meta-analysis
Source: BMC Psychiatry. 2017 Dec 21;17:406. doi: 10.1186/s12888-017-1575-9 (PMC5740861; doi:10.1186/s12888-017-1575-9)
Supplement: Supplementary file 2 — Quality of the eligible studies. Description of data: details about the quality of the eligible studies. (DOCX 17 kb) [file 12888_2017_1575_MOESM2_ESM.docx]

Quality of the eligible studies

| First author, year | Q1 | Q2 | Q3 | Q4 | Q5 | Q6 | Q7 | Q8 | Q9 | Q10 | Q11 | Score | Quality of study |
| --- | --- | --- | --- | --- | --- | --- | --- | --- | --- | --- | --- | --- | --- |
| Cochran, 2000 | Yes | Yes | Yes | Yes | No | Yes | No | UC | No | No | No | 5 | Moderate |
| Mathy, 2002 | Yes | Yes | Yes | Yes | No | No | Yes | No | No | UC | No | 5 | Moderate |
| Botnick, 2002 | Yes | Yes | Yes | No | No | Yes | No | No | No | No | Yes | 5 | Moderate |
| Warner, 2004 | Yes | Yes | Yes | No | No | UC | Yes | No | No | No | No | 4 | Moderate |
| de Graaf, 2006 | Yes | Yes | Yes | Yes | No | Yes | Yes | No | No | No | No | 6 | Moderate |
| Sheridan, 2009 | Yes | Yes | Yes | No | No | No | Yes | No | No | Yes | No | 5 | Moderate |
| Brennan, 2010 | Yes | Yes | Yes | Yes | No | No | No | No | UC | No | No | 4 | Moderate |
| Wang, 2012 | Yes | Yes | Yes | Yes | No | No | No | No | No | No | No | 4 | Moderate |
| Deuba, 2013 | Yes | Yes | Yes | No | No | Yes | No | No | No | No | No | 4 | Moderate |
| Wang, 2013 | Yes | Yes | Yes | Yes | No | No | No | No | No | Yes | Yes | 6 | Moderate |
| Chen, 2015 | Yes | UC | UC | No | No | No | No | No | No | Yes | No | 2 | Low |
| Ferlatte, 2015 | Yes | UC | Yes | No | No | No | No | No | UC | No | No | 2 | Low |
| Parker, 2015 | Yes | Yes | Yes | No | No | Yes | Yes | No | No | Yes | No | 6 | Moderate |
| Blosnich, 2016 | Yes | Yes | Yes | Yes | No | Yes | No | No | No | No | No | 5 | Moderate |
| Hottes, 2016 | Yes | Yes | Yes | Yes | No | No | No | No | No | No | No | 4 | Moderate |
| Mu, 2016 | Yes | Yes | Yes | No | No | Yes | No | No | No | No | No | 4 | Moderate |
| Hladik, 2016 | Yes | Yes | Yes | No | No | No | Yes | No | No | Yes | No | 5 | Moderate |
| Stahlman, 2016 | Yes | Yes | Yes | No | No | Yes | No | No | No | No | No | 4 | Moderate |
| Kohlbrenner, 2016 | Yes | Yes | Yes | No | No | Yes | No | Yes | No | No | No | 5 | Moderate |

UC: unclear

Q1: Define the source of information (survey, record review)

Q2: List inclusion and exclusion criteria for exposed and unexposed subjects (cases and controls) or refer to previous publications

Q3: Indicate time period used for identifying patients

Q4: Indicate whether or not subjects were consecutive if not population-based

Q5: Indicate if evaluators of subjective components of study were masked to other aspects of the status of the participants

Q6: Describe any assessments undertaken for quality assurance purposes (e.g., test/retest of primary outcome measurements)

Q7: Explain any patient exclusions from analysis

Q8: Describe how confounding was assessed and/or controlled.

Q9: If applicable, explain how missing data were handled in the analysis

Q10: Summarize patient response rates and completeness of data collection

Q11: Clarify what follow-up, if any, was expected and the percentage of patients for which incomplete data or follow-up was obtained
